# NK4, a four-kringle antagonist of HGF, inhibits spreading and invasion of human pancreatic cancer cells

**DOI:** 10.1054/bjoc.2000.1682

**Published:** 2001-03

**Authors:** N Maehara, K Matsumoto, K Kuba, K Mizumoto, M Tanaka, T Nakamura

**Affiliations:** Division of Biochemistry, Department of Oncology, Biomedical Research Center, Osaka University Graduate School of Medicine, Suita, Osaka, 565–0871, Japan; Department of Surgery and Oncology, Graduate School of Medical Sciences, Kyushu University, Maidashi, Fukuoka, 812–8582, Japan

**Keywords:** pancreatic cancer, HGF, HGF-antagonist, NK4, c-Met

## Abstract

Because of the highly aggressive behaviour, i.e. invasive, disseminative and metastatic properties, the outcome for patients with pancreatic cancer is morbid. A better understanding and interference with the malignant behaviour of pancreatic cancer may provide new directions for treatment. We report here the induction of highly motile and invasive properties in human pancreatic cancer cells by hepatocyte growth factor (HGF) and blockage of these properties by NK4, a newly identified antagonist for HGF. In all of eight human pancreatic cancer cell lines we used (AsPC-1, BxPC-3, H-48N, KP-1N, KP-2, KP-3, MIA PaCa-2 and SUIT-2 cells), the c-Met/HGF receptor was expressed at varying levels. Although weak mitogenic activity of HGF was seen only in SUIT-2 and KP-3 cells, HGF strongly stimulated migration and invasion of these pancreatic cancer cells, except for BxPC-3 and MIA PaCa-2 cells. In contrast, migration and invasion potently induced by HGF in KP-1N, KP-3 and SUIT-2 cells were inhibited by NK4. The invasion of SUIT-2 cells was also potently stimulated with the influence of cocultured pancreatic fibroblasts and by ascitic fluid obtained after pancreatic cancer resection, however, invasiveness of the cancer cells in such conditions was practically abolished by NK4. Consistently, the ascitic fluid in patients who had undergone pancreatic cancer surgery contained high levels of HGF. These findings mean that HGF is probably involved in invasion, dissemination, and metastasis of pancreatic cancer, particularly through tumour-stromal interaction and after resection of the pancreatic cancer. NK4, an effective antagonist of HGF, may prove to have the potential for anti-invasion/metastasis. © 2001 Cancer Research Campaign http://www.bjcancer.com


*British Journal of Cancer* (2001) **84**(6), 864–873
© 2001 Cancer Research Campaign
doi: 10.1054/ bjoc.2000.1682, available online at http://www.idealibrary.com on IDEAL®

### NK4, a four-kringle antagonist of HGF, inhibits spreading and invasion of human pancreatic cancer cells

**N Maehara[1,2], K Matsumoto[1], K Kuba[1], K Mizumoto[2], M Tanaka[2] and T Nakamura[1]**

[1]Division of Biochemistry, Department of Oncology, Biomedical Research Center, Osaka University Graduate School of Medicine, Suita, Osaka 565–0871, Japan; [2]Department of Surgery and Oncology, Graduate School of Medical Sciences, Kyushu University, Maidashi, Fukuoka 812–8582, Japan

**Summary** Because of the highly aggressive behaviour, i.e. invasive, disseminative and metastatic properties, the outcome for patients with pancreatic cancer is morbid. A better understanding and interference with the malignant behaviour of pancreatic cancer may provide new directions for treatment. We report here the induction of highly motile and invasive properties in human pancreatic cancer cells by hepatocyte growth factor (HGF) and blockage of these properties by NK4, a newly identified antagonist for HGF. In all of eight human pancreatic cancer cell lines we used (AsPC-1, BxPC-3, H-48N, KP-1N, KP-2, KP-3, MIA PaCa-2 and SUIT-2 cells), the c-Met/HGF receptor was expressed at varying levels. Although weak mitogenic activity of HGF was seen only in SUIT-2 and KP-3 cells, HGF strongly stimulated migration and invasion of these pancreatic cancer cells, except for BxPC-3 and MIA PaCa-2 cells. In contrast, migration and invasion potently induced by HGF in KP-1N, KP-3 and SUIT-2 cells were inhibited by NK4. The invasion of SUIT-2 cells was also potently stimulated with the influence of cocultured pancreatic fibroblasts and by ascitic fluid obtained after pancreatic cancer resection, however, invasiveness of the cancer cells in such conditions was practically abolished by NK4. Consistently, the ascitic fluid in patients who had undergone pancreatic cancer surgery contained high levels of HGF. These findings mean that HGF is probably involved in invasion, dissemination, and metastasis of pancreatic cancer, particularly through tumour-stromal interaction and after resection of the pancreatic cancer. NK4, an effective antagonist of HGF, may prove to have the potential for anti-invasion/metastasis. © 2001 Cancer Research Campaign http://www.bjcancer.com

**Keywords**: pancreatic cancer; HGF; HGF-antagonist; NK4; c-Met

Pancreatic cancer is one of the major causes of cancer-related deaths in industrialized countries (Poston et al, 1991; Warshaw and Castillo, 1992; Bramhall et al, 1995). Most pancreatic cancer patients were first diagnosed at an advanced stage of the disease and only 3% of all these patients survived for 5 years (Niederhuber et al, 1995). In addition to difficulties in an early diagnosis and lack of effective treatment, the aggressive behaviour of pancreatic cancer cells, i.e. potent invasive activity to the surrounding tissues and early metastatic ability to the distant organs, lead to a poor clinical outcome. Thus, a better understanding of mechanisms by which pancreatic cancers exhibit highly invasive and metastatic potential is needed for development of therapeutic intervention.

The invasive and metastatic ability of cancer depends on changes in adhesive properties of the cells, degradation of the extracellular matrix, and a concomitant induction of cell movement. Stromal cells in cancer tissues influence the growth, invasion and metastasis of cancer cells. Growth, migration, and invasion of cancer cells were markedly accelerated by a broad spectrum of fibroblasts in vivo (Camps et al, 1990) and in vitro (Grey et al, 1989). Over 90% of pancreatic cancers originate from pancreatic duct epithelial cells, accompanied by abundant stromal components. Therefore, local and mutual interactions between cancer cells and stromal cells are likely to be particularly important in regulating malignant behaviour in the pancreas.

Hepatocyte growth factor (HGF), initially identified and cloned as a potent mitogen for hepatocytes (Nakamura et al, 1984, 1989; Miyazawa et al, 1989), is a stromal-derived multi-potent factor that exhibits mitogenic, motogenic, and morphogenic activities. Accumulating evidence has shown that HGF plays a distinct role in tumour–stromal interactions (Seslar et al, 1993; Rosen et al, 1994; Matsumoto et al, 1996; Inoue et al, 1997; Nakamura et al, 1997; Jiang et al, 1999). HGF has potent motogenic activity on various types of carcinoma cells, leading to the dissociation, scattering and migration of cells (Weidner et al, 1990; Jiang et al, 1993, 1999; Matsumoto et al, 1994, 1996; Jeffers et al, 1996; Rosen et al, 1996; Inoue et al, 1997; Nakamura et al, 1997).

The c-Met/HGF receptor of membrane-spanning tyrosine kinase is expressed in a wide variety of carcinoma cells, including pancreatic cancer cells (Ebert et al, 1994; Di Renzo et al, 1995; Furukawa et al, 1995; Vila et al, 1995; Jiang et al, 1999). The invasion of pancreatic cancer cells was found to be accelerated with the addition of HGF with activation of the u-PA/u-PA receptor proteolytic system in vitro (Paciucci et al, 1998). Thus, functional coupling between HGF and the c-Met/HGF receptor in pancreatic cancer cells may play a key role in the invasion and metastasis of pancreatic cancer.

Based on the involvement of HGF on tumour malignancy, we earlier prepared an antagonist for HGF (Date et al, 1997). This HGF-antagonist, termed NK4, is composed of the N-terminal

*Received 27 April 2000*
*Revised 18 December 2000*
*Accepted 18 December 2000*

*Correspondence to:* T Nakamura; *Email*: nakamura@onbich.med.osaka-u.ac.jp hairpin domain and subsequent four kringle domains of the α-subunit of HGF. NK4 binds to the c-Met/HGF receptor, but does not induce tyrosine phosphorylation of c-Met (Date et al, 1997). While competitive inhibitory effects of NK4 on HGF and c-Met/receptor interaction have been demonstrated in some distinct types of human cancer cells (Date et al, 1998; Hiscox et al, 2000; Kuba et al, 2000; Parr et al, 2000), inhibitory and promising therapeutic effects of NK4 have to be evaluated in cases of highly aggressive pancreatic cancer. In the current study, cancer–stromal interaction through HGF and c-Met coupling and inhibitory effects of NK4 were investigated in human pancreatic cancer cells. The invasion of pancreatic cancer cells was potently stimulated by HGF, cocultivation with fibroblasts, and by ascitic fluid from patients who had undergone pancreatic cancer resection, but this invasion was almost completely inhibited by NK4. The potential inhibition of pancreatic cancer invasion and dissemination by NK4 was thus deemed worthy of investigation.

### MATERIALS AND METHODS

### Materials

Human recombinant HGF was purified from the conditioned medium of Chinese hamster ovary cells transfected with human HGF cDNA (Nakamura et al, 1989; Seki et al, 1990). Polyclonal antibody against human HGF was prepared from the serum of a rabbit immunized with human recombinant HGF and IgG was purified using protein A-Sepharose (Pharmacia Biotech, Uppsala). Anti-human HGF IgG (1 $\mu g\ ml^{-1}$) completely neutralized the biological activities of 1 ng $ml^{-1}$ human HGF. NK4 was prepared by proteolytic digestion with elastase, as described elsewhere (Date et al, 1997). Anti-c-Met polyclonal antibody (C-12) was obtained from Santa Cruz Biotechnology Inc. (Santa Cruz, CA). Human recombinant basic fibroblast growth factor (bFGF) and transforming growth factor-α (TGF-α) were obtained from R&D Systems (Minneapolis, MN).

### Cell culture

Eight human pancreatic cancer cell lines were used in this study: BxPC-3 and MIA PaCa-2 were provided by Japanese Cancer Resource Bank (Tokyo, Japan); AsPC-1, H-48N, KP-1N, KP-2, KP-3 and SUIT-2 were generously donated by Dr H Iguchi (National Kyushu Cancer Center, Fukuoka, Japan). Fibroblasts were initially proliferated outward from pancreatic tissue obtained at surgery, and used during 10–15 passages.

AsPC-1, H-48N, KP-1N, KP-2, KP-3 and SUIT-2 were cultured in RPMI supplemented with streptomycin, penicillin and 10% fetal bovine serum (FBS) at 37˚C in a humidified atmosphere containing 5% $CO_2$. BxPC-3, MIA PaCa-2 and fibroblasts were cultured in Dulbecco's modified Eagle's medium (DMEM) supplemented with streptomycin, penicillin and 10% FBS.

### Assay for cell growth and scattering

For measurement of cell proliferation, human pancreatic cancer cells in RPMI or DMEM with 10% FBS were respectively plated at $2.5 \times 10^3$ cells $cm^{-2}$ onto 24-well tissue culture plates and cultured for 24 h. The culture media were replaced by fresh RPMI or DMEM containing 5% FBS and the cells were cultured in absence or presence of 10 ng $ml^{-1}$ (110 pM) HGF for 72 h. The cells were also cultured with the addition of HGF in the absence or presence of 110 nM NK4. The number of cells was counted using a cell counter (Coulter, Bedfordshire) after dissociation with trypsin-EDTA (0.05% trypsin and 0.02% EDTA in PBS). For the cell-scattering assay, pancreatic cancer cells were plated at 250 cells $cm^{-2}$ in RPMI or DMEM containing 10% FBS onto six-well plates and cultured for 4–7 days. After the media were changed, 110 pM HGF, with or without 110 nM NK4, was added and after incubation for 24 h scattering of cells was microscopically observed.

### Migration assay

Migration of tumour cells was evaluated using a Transwell chamber (Corning Coster Co, Cambridge, MA) equipped with a filter membrane with 8-μm pores. Cancer cells were plated at $5 \times 10^4$ cells $cm^{-2}$ or $1 \times 10^4$ cells $cm^{-2}$ in RPMI or DMEM containing 10% FBS onto the upper compartment of the chamber. Cells were cultured in the absence or presence of HGF for 24 h, fixed in 70% ethanol and stained with haematoxylin and eosin. Cells migrating to the undersurface of the membrane through the pores, as seen microscopically, were counted. Five microscopic fields (× 200) were randomly selected for cell counting. To examine the inhibitory effects on migration of tumour cells, NK4 and antibody against HGF were added to the lower compartment. The migration assays were independently performed three times and similar results were obtained in each experiment.

### Invasion assay

Invasion of tumour cells was measured using a 24-well Matrigel invasion chamber (Becton Dickinson, Bedford, MA). The pancreatic cancer cells suspended in RPMI or DMEM containing 2% FBS were added to the inner cup of a Matrigel invasion chamber at a density of $1 \times 10^5$ cells $cm^{-2}$. HGF or ascitic fluid, and/or NK4 were added to the medium of the outer cup. After 24 h cultivation, cells that degraded the Matrigel and migrated through 8-μm pores of the membrane to the opposite side of the membrane were counted after they had been stained with haematoxylin and eosin.

For cocultivation of pancreatic cancer cells and fibroblasts, human fibroblasts were initially seeded on the outer cup of a Matrigel invasion chamber at a density of $1.5 \times 10^4$ cells $cm^{-2}$ and cultured in DMEM containing 10% FBS for 24 h. The medium was replaced by fresh medium supplemented with 2% FBS, and pancreatic cancer cells were seeded on the inner cup of the invasion chamber at a density of $1 \times 10^5$ cells $cm^{-2}$ and cultured for 24 h. After cocultivation, cells which passed through the membrane were counted. The invasion assays were independently performed three times and similar results were obtained in each experiment.

### Western immunoblotting of the c-Met/HGF receptor

After reaching confluency, carcinoma cells were scraped, collected by centrifugation, and solubilized in 500 μl of ice-cold lysis buffer composed of 150 mM NaCl, 20 mM Tris-HCl (pH 7.5), 10 mM EDTA, 5 $\mu g\ ml^{-1}$ leupeptin, 1 mM phenylmethy sulfonyl fluoride and 0.5% (v/v) Triton X-100. The supernatants were collected by centrifugation and cell lysates were subjected to

© 2001 Cancer Research Campaign

SDS-PAGE at 100 μg protein $lane^{-1}$, under reducing conditions, using a 6% polyacrylamide gel. The proteins were transferred to a polyvinylidene difluoride membrane (Bio-Rad, Hercules, CA), and the membrane was incubated with anti-human Met antibody, biotinylated goat anti-rabbit IgG (Vector, Burlingame, CA), and the peroxidase-conjugated avidin–biotin complex. The c-Met/HGF receptor was visualized using an ECL enhanced chemiluminescence method (Amersham, Little Chalfont).

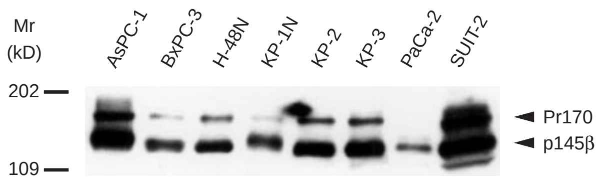

**Figure 1** Expression of c-Met/HGF receptor in human pancreatic cancer cell lines. The cells were solubilized in lysis buffer and the proteins were electrophoresed on SDS-PAGE, under reducing conditions. The proteins were transferred onto the membrane and probed with antibodies against the c-Met/HGF receptor

### Collection of ascitic fluid and measurement of HGF

Ascitic fluid was obtained from peritoneal drain tubes from three Japanese patients with pancreatic cancer who had undergone resection for primary pancreatic cancer in the Department of Surgery and Oncology, Graduate School of Medical Sciences, Kyushu University. The present study was carried out with the approval of the ethical committee organized by the senior staff in the Department of Surgery and Oncology. Written informed consent for collecting ascitic fluid samples for research was obtained from each patient prior to the surgery. The supernatants of the samples, collected by centrifugation at 2200 rpm for 20 min, were stored at –80˚C until assay.

HGF concentration in the conditioned media and the ascitic fluid was measured using an enzyme-linked immunosorbent assay (ELISA) kit (Institute of Immunology, Tokyo), according to the manufacturer's instructions. The lower detection limit of this assay is 0.10 ng $ml^{-1}$.

### RESULTS

### Expression of c-Met/HGF receptor

We first analysed expression of the c-Met/HGF receptor in human pancreatic carcinoma cells by Western immunoblotting. The β subunit ($M_r$ 145 000, p 145 β) of c-Met/HGF receptor was identified in all carcinoma cell lines and the precursor form of c-Met/HGF receptor ($M_r$ 170 000, $P_r$ 170) was identified in seven cell lines but not PaCa-2 (Figure 1). These pancreatic cancer cells expressed varying levels of c-Met/HGF receptor: relatively strong expression was seen in AsPC-1 and SUIT-2 cells, and medium or low expression was seen in BxPC-3, H-48N, KP-1N, KP-2 and KP-3 cells. MIA PaCa-2, which did not show any response to HGF (see below) had the weakest expression of the β subunit and no expression of the precursor of the c-Met/HGF receptor. Thus, eight distinct types of human pancreatic cancer cell lines expressed varying levels of c-Met/HGF receptor, which means that these cancer cells are potential target cells of HGF.

### NK4 inhibits HGF-induced cell growth and scattering

We next analysed effects of HGF on proliferation of pancreatic cancer cells (Table 1). With the addition of 10 ng $ml^{-1}$ (110 pM) HGF, proliferation of KP-3 and SUIT-2 pancreatic cancer cells were stimulated by 1.4-fold (Table 1). However, cell proliferation was not affected in other lines. On the other hand, stimulatory effects of 110 pM HGF on proliferation of the KP-3 and SUIT-2 cells was almost completely inhibited in the presence of 110 nM NK4 (not shown).

As HGF is a potent stimulator of epithelial cell colony dissociation, which results in scattering of cells (Gherardi et al, 1989; Weidner et al, 1990), we tested the effects of HGF on scattering of pancreatic cancer cells in monolayer culture (Figure 2A). Six pancreatic cancer cells (BxPC-3, H-48N, KP-1N, KP-2, KP-3 and SUIT-2) formed colonies, displaying varying levels of intercellular adhesions. The addition of 10 ng $ml^{-1}$ HGF led to dissociation of the colonies and stimulated motility, resulting in scatterings of KP-2, KP-3 and SUIT-2 cells. Although HGF did not cause a remarkable dissociation of colonies in H-48N and KP-1N lines, these cells had a flattened and spindle-like appearance when HGF was added, and several cells were scattered, thereby suggesting that

**Table 1** c-Met/HGF receptor expression, HGF production and biological effects of HGF on pancreatic cancer cells

| Pancreatic cancer cell lines | c-Met/HGF receptor expression | HGF production | Biological effects | | |
|---|---|---|---|---|---|
| | | | Growth[a] | Migration[b] | Invasion[b] |
| AsPC-1 | +++ | undetectable | no effect | +++ | +++ |
| BxPC-3 | + | 0.1 ng $ml^{-1}$ | no effect | no effect | no effect |
| H-48N | ++ | 0.1 ng $ml^{-1}$ | no effect | +++ | ++ |
| KP-1N | + | 0.2 ng $ml^{-1}$ | no effect | +++ | ++ |
| KP-2 | ++ | 0.1 ng $ml^{-1}$ | no effect | +++ | +++ |
| KP-3 | ++ | 0.1 ng $ml^{-1}$ | 1.4 | +++ | +++ |
| PaCa-2 | + | undetectable | no effect | no effect | no effect |
| SUIT-2 | +++ | undetectable | 1.4 | +++ | +++ |

[a]Stimulation in fold increase; [b]++ = stimulation in 2–3-fold increase; +++ = > 3-fold increase

 © 2001 Cancer Research Campaign

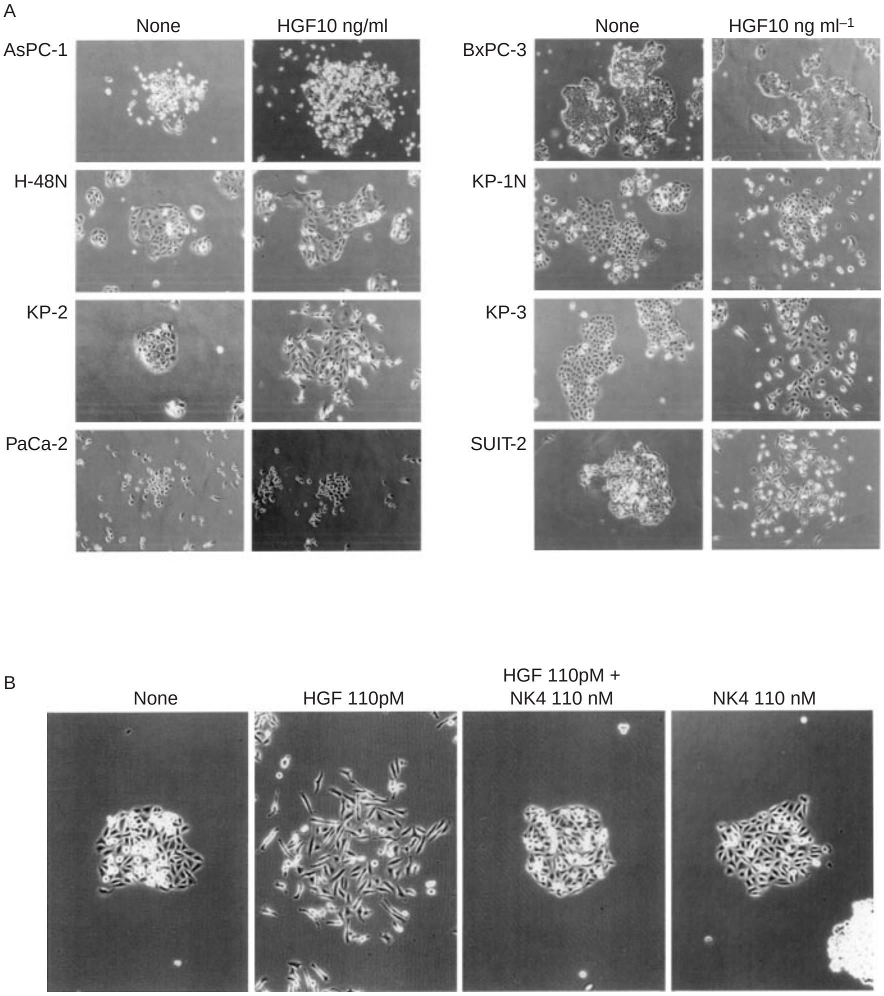

**Figure 2** (**A**) Enhancement of cell scattering of human pancreatic cancer cells by HGF, (**B**) inhibition of HGF-induced cell scattering by NK4 in SUIT-2 cells. Pancreatic cancer cells were cultured for 24 h in the presence of 10 ng $ml^{-1}$ (110 pM) HGF and/or 110 nM NK4.

HGF weakly stimulated the motility of these cells. Cell scattering was not induced by HGF in BxPC-3 cells. AsPC-1 and MIA PaCa-2 cells seemed to form loose cell–cell interactions, showing relatively scattered appearances even without HGF and no remarkable change was observed even after the addition of HGF. To determine if NK4 would inhibit HGF-induced scattering of pancreatic cancer cells, effects of NK4 on KP-2, KP-3 and SUIT-2 cells were examined. As shown in Figure 2B, cell scattering of SUIT-2 cells induced by 110 pM HGF was completely blocked by 110 nM NK4, whereas NK4 alone had no effect on the cell scattering. Similar inhibitory effects of NK4 on HGF-induced cell scattering were seen in KP-2 and KP-3 lines (not shown).

### NK4 inhibits migration and invasion of pancreatic cancer cells

We measured the migration of carcinoma cells in vitro using a Transwell chamber. In the absence of HGF, the number of cells migrating through pores to the opposite side of the membrane was 20 $field^{-1}$ or fewer. In the presence of 10 ng $ml^{-1}$ HGF, migration of the cells was evidently stimulated in AsPC-1, H-48N, KP-1N, KP-2, KP-3 and SUIT-2 lines by 3.4-fold, 5.5-fold, 5.1-fold, 10.5-fold, 6.3-fold and 13.7-fold, respectively, compared to cultures without HGF (Figure 3A). Although HGF stimulated migration of these cells, a small number of cells migrated even in the presence of HGF in AsPC-1, BxPC-3, and H-48N cells. When migration of these cells was examined in the presence of other growth factors, bFGF and TGF-α but not TGF-β and platelet-derived growth factor weakly stimulated migration of AsPC-1 cells, whereas these growth factors had no obvious effect on migration of BxPC-3 and H-48N cells (not shown). Therefore, these cells seem to have a general poor motility, compared to other cells. On the other hand, migration of the cells was not significantly stimulated in BxPC-3 and MIA PaCa-2 by HGF. We then examined effects of NK4 on HGF-induced migration of SUIT-2 cells, which showed an evident responsiveness to HGF. NK4 inhibited the stimulatory effects of HGF on migration of SUIT-2 cells, in a dose-dependent manner (Figure 3B): a significant inhibition of migration was seen at 0.33 nM NK4, a 10-fold higher concentration than that of HGF (33 pM) and 33 nM of NK4 prohibited the HGF-induced migration of

© 2001 Cancer Research Campaign

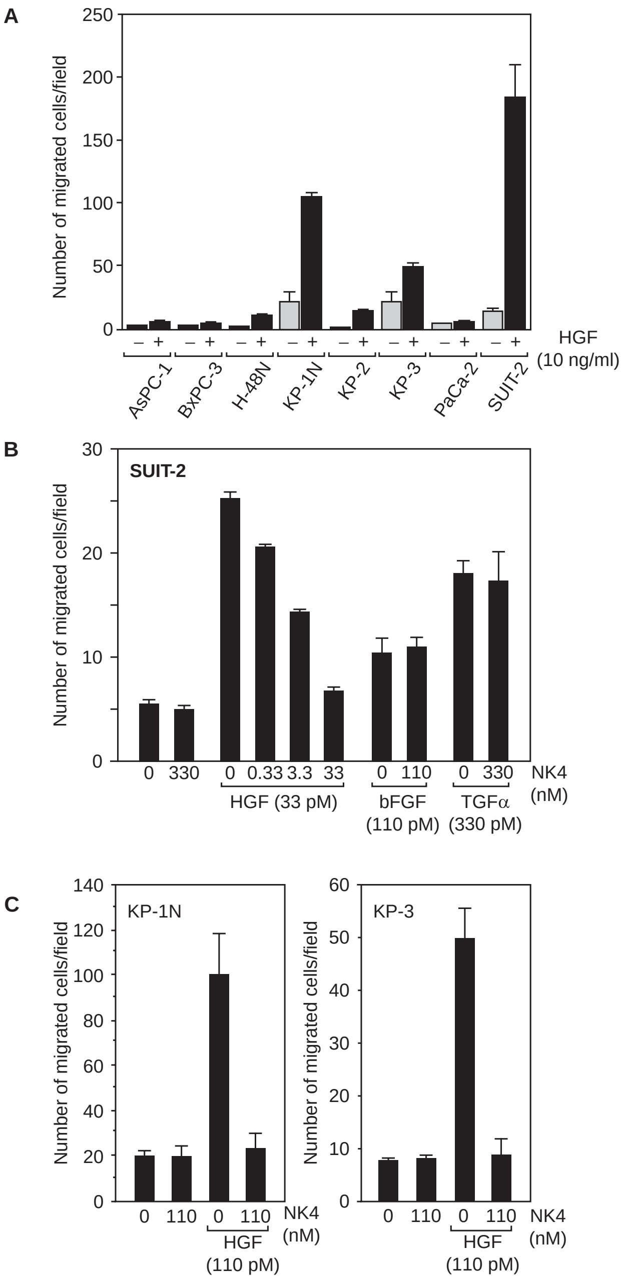

**Figure 3** (**A**) Enhancement of migration of human pancreatic cancer cells by HGF, (**B**) inhibitory effect of NK4 on migration of SUIT-2, (**C**) KP-1N and KP-3 cells. Cells were plated at $5 \times 10^4$ cells cm$^{-2}$ (**A, C**) or $1 \times 10^4$ cells cm$^{-2}$ (**B**). In (**A**), pancreatic cancer cells were cultured on a Transwell chamber for 24 h in the absence or presence of HGF. In (**B**), SUIT-2 cells were cultured for 24 h in the presence of HGF, bFGF and TGF-α, with or without NK4. In (**C**), KP-1N and KP-3 cells were cultured for 24 h in the presence of HGF, with or without NK4. Cells migrating through 8-μm pores of the filter membrane to the underside of the membrane were counted. Each value represents the mean ± SD of triplicate measurements

SUIT-2 cells. However, 330 nM NK4 alone had no effect on migration of SUIT-2 cells. On the other hand, bFGF and TGF-α also stimulated migration of the cells, whereas NK4 did not inhibit migration of the cells stimulated by bFGF and TGF-α (Figure 3B). Similarly, 110 pM HGF stimulated migration of KP-1N and KP-3 cells, but the migration of these cells enhanced by HGF was almost completely inhibited by 110 nM NK4 (Figure 3C).

To determine if HGF would affect invasion of pancreatic carcinoma cells, cells were cultured in a Matrigel invasion chamber in the absence or presence of HGF. Except for BxPC-3 and MIA PaCa-2 cells, HGF stimulated invasion of pancreatic cancer cells. HGF (10 ng ml$^{-1}$) potently stimulated the invasion of KP-1N, KP-2, KP-3 and SUIT-2 cells by 2.1-fold, 3.4-fold, 4.4-fold, and 4.7-fold (Figure 4A), respectively. In cases of AsPC-1 and H-48N cells, the number of invasive cells was few, but HGF did stimulate invasion of these cells. Therefore, HGF is a potent stimulator of invasion in many but not all pancreatic cancer cells. Inhibitory effects of NK4 on tumour cell invasion were then examined using SUIT-2, KP-1N and KP-3 cells. In the absence of HGF, the number of invading cells was fewer than 20 field$^{-1}$ (Figure 4B). The addition of 110 pM HGF strongly stimulated the invasion of SUIT-2 cells through the Matrigel membrane, whereas NK4 dose-dependently blocked the invasion induced by HGF. The invasion stimulated by 110 pM HGF was significantly inhibited by 1.1 nM NK4 and was almost completely inhibited by 110 nM NK4, a 1000-fold higher concentration of HGF. The addition of 110 nM NK4 alone had no apparent effect on invasion of the cells. Similarly, 110 nM NK4 inhibited invasion of KP-1N and KP-3 cells enhanced by 110 pM HGF, while NK4 alone had no effect on invasion of these cells (Figure 4C).

Since invasive behaviour of carcinoma cells is regulated by interactions with stromal fibroblasts, we asked if the invasive potential of SUIT-2 pancreatic cancer cells is regulated by interaction with pancreatic fibroblasts. SUIT-2 cells were cultured on the Matrigel, while fibroblasts were cultured in the outer well. When SUIT-2 cells were cultured alone without fibroblasts, the number of SUIT-2 cells which invaded through Matrigel and the membrane was below 10 cells per field, as shown in Figure 5. In contrast, SUIT-2 cells markedly invaded when they were cultured with fibroblasts: the number of invaded cells increased by 6.6-fold by cocultivation with fibroblasts. Importantly, NK4 dose-dependently blocked the invasion of SUIT-2 cells in the presence of cocultured fibroblasts. Complete inhibition by NK4 was seen with 110 nM. The addition of antibody against HGF also inhibited the invasion stimulated by fibroblasts (Figure 5). These results indicate that: 1. the invasive potential of SUIT-2 cells is strongly enhanced by interaction with stromal fibroblasts; 2. the fibroblast-derived factor which enhances SUIT-2 invasion is HGF; and 3. NK4 abrogates SUIT-2–fibroblasts interaction as mediated by HGF, and thereby inhibits SUIT-2 invasion.

### NK4 inhibits cancer invasion stimulated by ascitic fluid after resection

Since surgical resection of carcinomas often results in acceleration of cancer progression and dissemination (Kodama et al, 1992; Bogden et al, 1997), we considered that HGF might influence the invasive potential of pancreatic cancers after surgery. To address this issue, SUIT-2 cells were cultured in the Matrigel invasion chamber in the presence of ascites obtained from a surgical patient with pancreatic cancer (Figure 6A). As demonstrated above, invasion of SUIT-2 cells was strongly stimulated by the addition of 110

 *© 2001 Cancer Research Campaign*

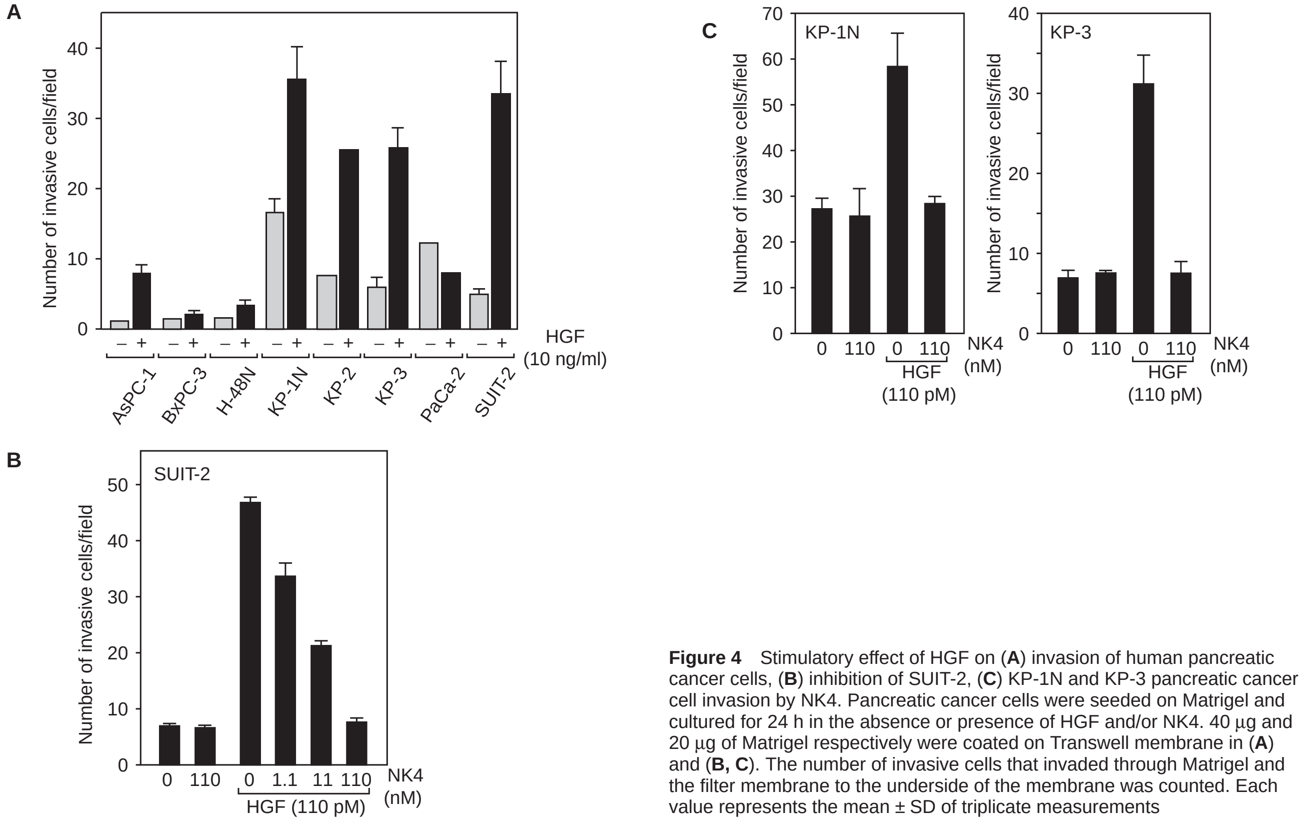

**Figure 4** Stimulatory effect of HGF on (**A**) invasion of human pancreatic cancer cells, (**B**) inhibition of SUIT-2, (**C**) KP-1N and KP-3 pancreatic cancer cell invasion by NK4. Pancreatic cancer cells were seeded on Matrigel and cultured for 24 h in the absence or presence of HGF and/or NK4. 40 µg and 20 µg of Matrigel respectively were coated on Transwell membrane in (**A**) and (**B, C**). The number of invasive cells that invaded through Matrigel and the filter membrane to the underside of the membrane was counted. Each value represents the mean ± SD of triplicate measurements pM HGF. Likewise, the addition of ascitic fluid strongly stimulated the invasion of SUIT-2 cells and the maximal activity of ascites to stimulate cancer invasion was much higher than that of 110 pM HGF. The potent stimulatory effect on SUIT-2 cell invasion was also evident in ascites obtained from other patients (not shown). Therefore, the ascitic fluid obtained after surgery of pancreatic cancer contains a factor(s) which stimulates the invasion of SUIT-2 cells.

To determine if HGF is involved in the potent ability of ascitic fluid to stimulate tumour invasion, HGF levels in ascites were determined using ELISA. The HGF level in sera of healthy volunteers was 0.36 ± 0.13 ng $ml^{-1}$ (Uchiyama et al, 1999), while levels in ascitic fluid obtained after pancreatic cancer surgery were much higher. The mean ascitic HGF level obtained 2 days after surgery reached 7.0 ng $ml^{-1}$ (Figure 6B), then the level gradually decreased.

Whether or not NK4 inhibits tumour invasion stimulated by ascites obtained after pancreatic cancer surgery was also determined. The addition of the ascites strongly stimulated the invasion of SUIT-2 cells, whereas the invasion stimulated by the ascites was dose-dependently inhibited by NK4 and the highest concentration of NK4 (110 nM) almost completely inhibited invasion of SUIT-2 cells (Figure 6C). We propose that HGF in ascitic fluid is responsible for the potent activity required to stimulate invasion.

### DISCUSSION

The identification of potential targets for therapeutic intervention in the cancer patient will be aided by a better understanding of molecular and cellular mechanisms which underlie tumour invasion and metastasis. Previous studies and our present study indicate that the c-Met/HGF receptor is expressed in not all but in many distinct types of human pancreatic cancer cell lines and pancreatic cancer tissues, and that HGF affects growth, locomotion and invasive behaviour of pancreatic cancer cells (Ebert et al, 1994; Di Renzo et al, 1995; Furukawa et al, 1995; Paciucci et al, 1998). Our results particularly strengthen the notion of involvement of HGF in invasive potential rather than growth potential in human pancreatic cancers. Since previous (Paciucci et al, 1998) and our present results showed that production of HGF in pancreatic cancer cell lines is either undetectable or is low and that HGF mediated fibroblast-dependent invasion of pancreatic cancer cells, HGF is likely to be a stromal mediator which affects invasion and probably subsequent metastasis and dissemination of pancreatic cancer cells. Taken together with the notion that HGF is a potent inducer of angiogenesis (Bussolino et al, 1992; Van Belle et al, 1998; Morishita et al, 1999), the abrogation of functional association between HGF and the Met/HGF receptor would be considerable to suppress malignant behaviour of human pancreatic cancer cells.

Although molecular mechanisms by which HGF exhibits profound effects on tumour cell invasion have not been fully defined, it is known that HGF activates intracellular and extracellular events that lead to dissociation and invasion of cancer cells. HGF decreases cadherin-mediated adhesiveness (Watabe et al, 1993; Shibamoto et al, 1994; Tannapfel et al, 1994; Nabeshima et al, 1998), enhances cell-matrix interaction through the recruitment of integrins, $p125^{FAK}$, and paxillin into focal adhesion complexes and concomitant protein phosphorylation of $p125^{FAK}$

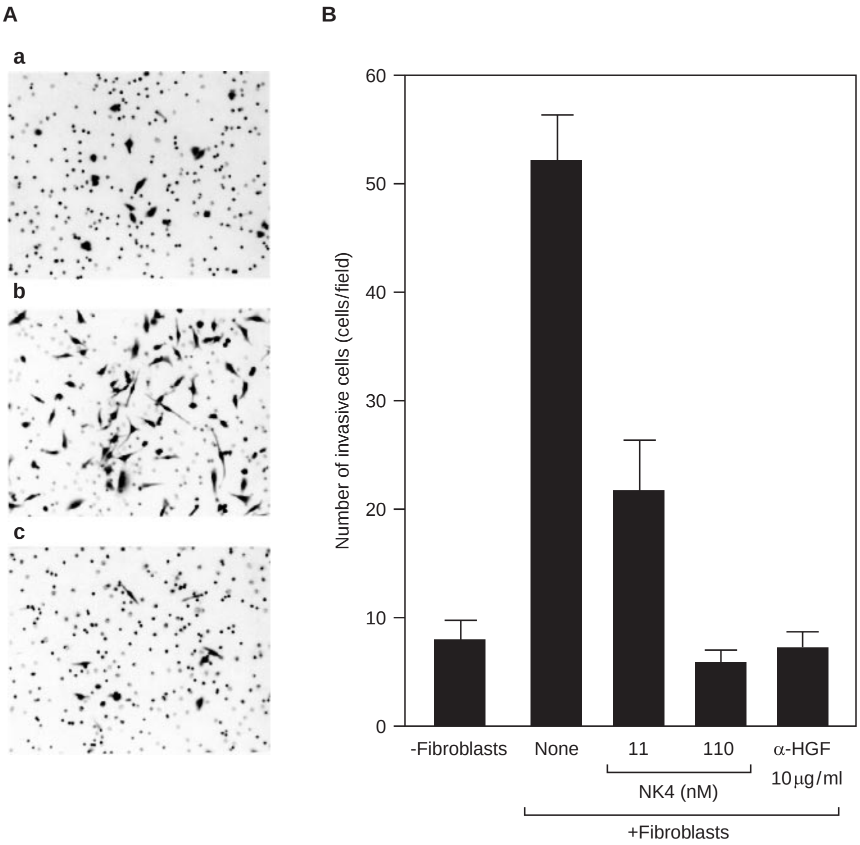

**Figure 5** Inhibitory effect of NK4 on invasion of SUIT-2 pancreatic cancer cells in coculture with fibroblasts. (**A**) Appearance of invaded SUIT-2 cells. SUIT-2 cells were cultured for 24 h without fibroblasts (**a**), or were cocultured with fibroblasts in the absence (**b**) or presence of 110 nM NK4 (**c**). 20 μg of Matrigel was coated on the Transwell membrane. (**B**) The number of cells that invaded through Matrigel and the filter membrane. SUIT-2 cells were cultured for 24 h without fibroblasts, or they were cocultured with fibroblasts in the absence or presence of 11 nM or 110 nM NK4. Each value represents the mean ± SD of triplicate measurements and paxillin (Matsumoto et al, 1994; Jiang et al, 1996). HGF stimulates proteolytic breakdown of the extracellular matrix, through enhancing matrix methaloproteinases (MMPs) and urokinase-type plasminogen activator (uPA)-dependent proteolytic network (Pepper et al, 1992; Jeffers et al, 1996; Date et al, 1998; Kadono et al, 1998; Rosenthal et al, 1998). On the other hand, we found that NK4 inhibits scattering, migration and invasion of SUIT-2 cells, induced by HGF, cocultured fibroblasts and ascitic fluid obtained from patients who underwent pancreatic cancer surgery. NK4 binds to the c-Met/ HGF receptor with a 10-fold lower affinity than HGF, while tyrosine phosphorylation of the c-Met/HGF receptor was almost completely inhibited by NK4 at 1000-fold higher concentration than that of HGF (Date et al, 1997, 1998; Kuba et al, 2000). NK4 dose-dependently inhibited pancreatic cancer cell migration and invasion, which coincides with the competitive inhibition of HGF-binding to the Met receptor by NK4. NK4 inhibits MMP-9 and uPA activities stimulated by HGF and NK4 inhibits migration and invasion of distinct types of cancer cells, including gallbladder, colon and breast cancer cells (Date et al, 1998; Hiscox et al, 2000; Parr et al, 2000). It is likely that NK4 inhibits HGF-dependent intracellular and extracellular events that accelerate the dissociation and invasion in pancreatic cancer cells.

Acceleration of cancer progression after surgery is evident in clinical settings and in experimental animals (Kodama et al, 1992; Bogden et al, 1997). This means that tissue injury accompanying surgical treatment may offer the specific environment which influences dissemination of cancer cells, if residual cancer cells are present in the resected margin or in the wound fluid. In pancreatic cancer treatment, disseminative cancer spreading and metastasis often follows surgical removal of the primary tumour (Sperti et al, 1997). We found that high levels of HGF exist in peritoneal fluid after pancreatic cancer surgery and that incubation with ascitic fluid strongly stimulates invasion of pancreatic cancer cells, yet this invasion was almost completely inhibited by an HGF-antagonist and neutralizing HGF antibody. This finding suggests that HGF may be involved in the aggressive invasion, dissemination, and/or metastasis of postoperative pancreatic cancer. Previous studies also noted that HGF is present in most pleural and peritoneal fluid after lung and hepatic surgery respectively (Eagles et al, 1996; Kimura et al, 1996; Uchiyama et al, 1999). Although tissue and serum HGF levels rapidly increase after tissue injuries and HGF plays an important role in tissue regeneration (Zarnegar and Michalopoulos, 1995; Matsumoto and Nakamura, 1997), in cancer tissues, HGF in the wound fluid may accelerate spreading and dissemination of remnant cancer cells. Inhibition

 *© 2001 Cancer Research Campaign*

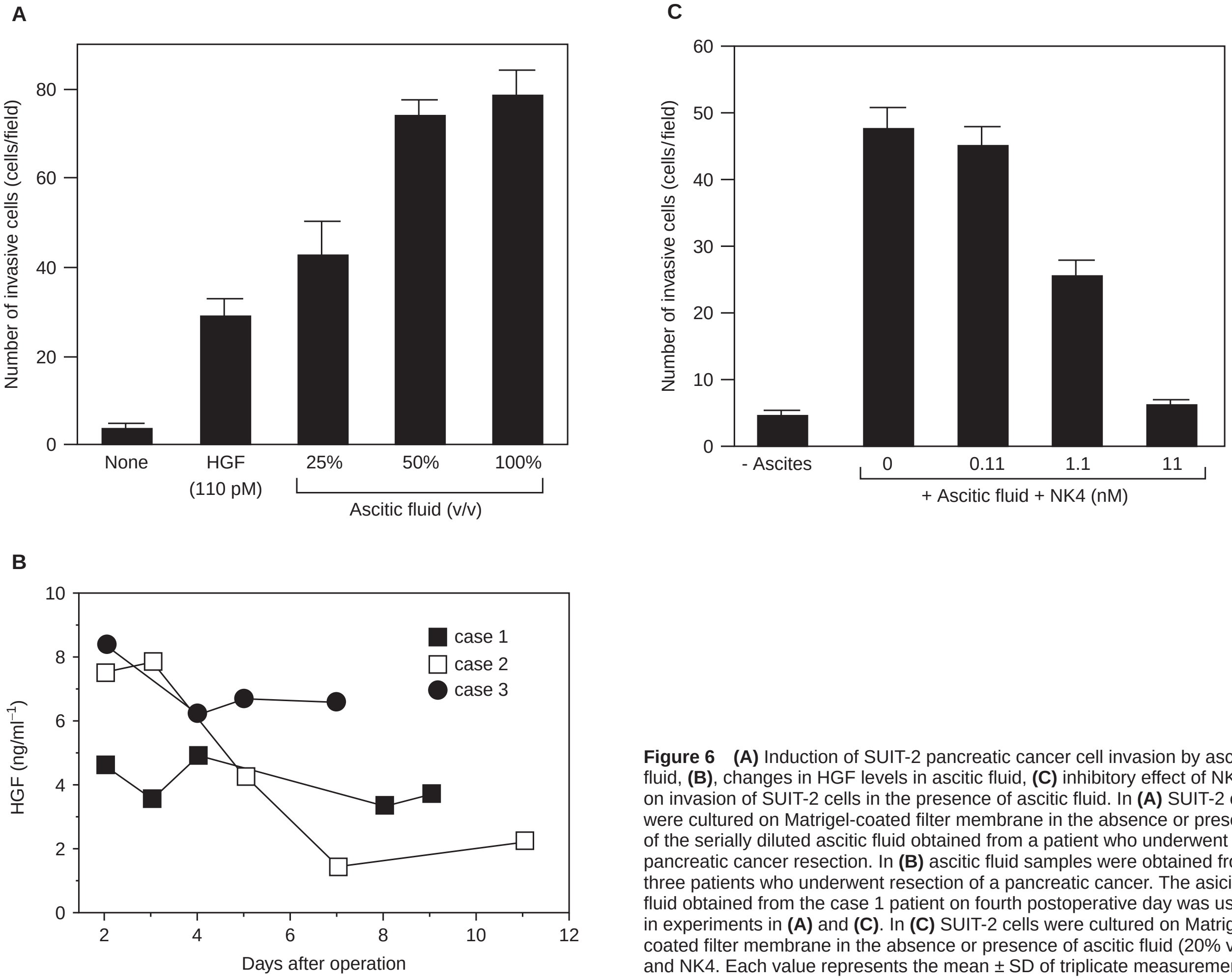

**Figure 6** **(A)** Induction of SUIT-2 pancreatic cancer cell invasion by ascitic fluid, **(B)**, changes in HGF levels in ascitic fluid, **(C)** inhibitory effect of NK4 on invasion of SUIT-2 cells in the presence of ascitic fluid. In **(A)** SUIT-2 cells were cultured on Matrigel-coated filter membrane in the absence or presence of the serially diluted ascitic fluid obtained from a patient who underwent pancreatic cancer resection. In **(B)** ascitic fluid samples were obtained from three patients who underwent resection of a pancreatic cancer. The asicitic fluid obtained from the case 1 patient on fourth postoperative day was used in experiments in **(A)** and **(C)**. In **(C)** SUIT-2 cells were cultured on Matrigel-coated filter membrane in the absence or presence of ascitic fluid (20% v/v) and NK4. Each value represents the mean ± SD of triplicate measurements of ascites-induced invasion of pancreatic cancer cells by NK4 suggests the possibility that NK4 may be applicable to treat pancreatic cancer patients who have had surgery.

In conclusion, HGF potently stimulates dissociation and the invasive potential of pancreatic cancer cells and is likely to be involved in invasion, dissemination and metastasis of pancreatic cancers, possibly through tumour–stromal interaction and also after pancreatic cancer surgery. NK4, an effective antagonist of HGF-induced invasive and metastatic behaviour, may have the potential for anti-invasion/metastatic therapy.

### ACKNOWLEDGEMENTS

We thank Dr N Taniura (Department of Oncology, Osaka University) for technical assistance and M Ohara for helpful comments. This study was supported in part by a Grant-in-Aid from the Ministry of Education, Science, Sports and Culture of Japan, Tokyo Biochemical Research Foundation, Research Grants from Nissan Science Foundation, Princess Takamatsu Cancer Research Fund, Takeda Science Foundation, the Pancreas Research Foundation of Japan and Kyushu University Interdisciplinary Programs in Education and Projects in Research Development.

### REFERENCES

Bogden AE, Moreau JP and Eden PA (1997) Proliferative response of human and animal tumours to surgical wounding of normal tissues: onset, duration and inhibition. *Br J Cancer* **75**: 1021–1027

Bramhall SR, Allum WH, Jones AG, Allwood A, Cummins C and Neoptolemos JP (1995) Treatment and survival in 13 560 patients with pancreatic cancer, and incidence of the disease, in the West Midlands: an epidemiological study. *Br J Surg* **82**: 111–115

Bussolino F, Di Renzo MF, Ziche M, Bocchietto E, Olivero M, Naldini L, Gaudino G, Tamagnone L, Coffer A and Comoglio PM (1992) Hepatocyte growth factor is a potent angiogenic factor which stimulates endothelial cell motility and growth. *J Cell Biol* **119**: 629–641

Camps JL, Chang S, Hsu TC, Freeman MR, Hong S, Zhau HE, von Eschenbach AC and Chung LW (1990) Fibroblast-mediated acceleration of human epithelial tumor growth in vivo. *Proc Natl Acad Sci USA* **87**: 75–79

Date K, Matsumoto K, Shimura H, Tanaka M and Nakamura T (1997) HGF/NK4 is a specific antagonist for pleiotrophic actions of hepatocyte growth factor. *FEBS Lett* **420**: 1–6

Date K, Matsumoto K, Kuba K, Shimura H, Tanaka M and Nakamura T (1998) Inhibition of tumor growth and invasion by a four-kringle antagonist (HGF/NK4) for hepatocyte growth factor. *Oncogene* **17**: 3045–3054

Di Renzo MF, Poulsom R, Olivero M, Comoglio PM and Lemoine NR (1995) Expression of the Met/hepatocyte growth factor receptor in human pancreatic cancer. *Cancer Res* **55**: 1129–1138

Eagles G, Warn A, Ball RY, Baillie-Johnson H, Arakaki N, Daikuhara Y and Warn RM (1996) Hepatocyte growth factor/scatter factor is present in most pleural effusion fluids from cancer patients. *Br J Cancer* **73**: 377–381

© 2001 Cancer Research Campaign 

Ebert M, Yokoyama M, Friess H, Buchler MW and Korc M (1994) Coexpression of the c-met proto-oncogene and hepatocyte growth factor in human pancreatic cancer. *Cancer Res* **54**: 5775–5778

Furukawa T, Duguid WP, Kobari M, Matsuno S and Tsao MS (1995) Hepatocyte growth factor and Met receptor expression in human pancreatic carcinogenesis. *Am J Pathol* **147**: 889–895

Gherardi E, Gray J, Stoker M, Perryman M and Furlong R (1989) Purification of scatter factor, a fibroblast-derived basic protein that modulates epithelial interaction and movement. *Proc Natl Acad Sci USA* **86**: 5844–5848.

Grey AM, Schor AM, Rushton G, Ellis I and Scholr SL (1989) Purification of the migration stimulating factor produced by fetal and breast cancer patient fibroblasts. *Proc Natl Acad Sci USA* **86**: 2438–2442

Hiscox S, Parr C, Nakamura T, Matsumoto K, Mansel RE and Jiang WG (2000) Inhibition of HGF/SF-induced breast cancer cell motility and invasion by the HGF/SF variant, NK4. *Breast Cancer Res & Treat* **1627**: 1–10

Inoue T, Chung YS, Yashiro M, Nishimura S, Hasuma T, Otani S and Sowa M (1997) Transforming growth factor-beta and hepatocyte growth factor produced by gastric fibroblasts stimulates the invasiveness of scirrhous gastric cancer cells. *Jpn J Cancer Res* **88**: 152–159

Jeffers M, Rong S and Vande Woude GF (1996) Enhanced tumorigenicity and invasion-metastasis by hepatocyte growth factor/scatter factor-met signalling in human cells concomitant with induction of the urokinase proteolysis network. *Mol Cell Biol* **16**: 1115–1125

Jiang WG, Lloyds D, Puntis MCA, Nakamura T and Hallet MB (1993) Regulation of spreading and growth of colon cancer cells by hepatocyte growth factor. *Clin Exp Metastasis* **11**: 235–242

Jiang WG, Hiscox S, Nakamura T, Hallett MB, Puntis MCA and Mansel RE (1996) Hepatocyte growth factor/scatter factor induces tyrosine phosphorylation of focal adhesin kinase (FAK) and paxillin and enhances cell-matrix interactions. *Oncology Rep* **3**: 819–823

Jiang WG, Hiscox S, Matsumoto K and Nakamura T (1999) Hepatocyte growth factor/scatter factor, its molecular, cellular, and clinical implications in cancer. *Crit Rev Oncol Hematol* **29**: 209–248

Kadono Y, Shibahara K, Namiki M, Watanabe Y, Seiki M and Sato H (1998) Membrane type-matrix metalloproteinase is involved in the formation of hepatocyte growth factor/scatter factor-induced branching tubules in Mardin-Darby canine kidney epithelial cells. *Biochem Biophys Res Commun* **251**: 681–687

Kimura F, Miyazaki M, Suwa T, Kakizaki S, Itoh H, Kaiho T, Ambiru S, Shimizu H and Togawa A (1996) Increased levels of human hepatocyte growth factor in serum and peritoneal fluid after partial hepatectomy. *Am J Gastroenterol* **91**: 116–121

Kodama M, Kodama T, Nishi Y and Totani R (1992) Does surgical stress cause tumor metastasis? *Anticancer Res* **12**: 1603–1616

Kuba K, Matsumoto K, Date K, Shimura H, Tanaka M and Nakamura T (2000) HGF/NK4, a four-kringle antagonist of hepatocyte growth factor, is an angiogenesis inhibitor that suppresses tumor growth and metastasis in mice. *Cancer Res* **60**: 6737–6743

Matsumoto K, Matsumoto K, Nakamura T and Kramer RH (1994) Hepatocyte growth factor/scatter factor induces tyrosine phosphorylation of focal adhesion kinase ($p125^{FAK}$) and promotes migration and invasion by oral squamous cell carcinoma cells. *J Biol Chem* **269**: 31807–31813

Matsumoto K, Date K, Shimura H and Nakamura T (1996) Acquisition of invasive phenotype in gallbladder cancer cells via mutual interaction of stromal fibroblasts and cancer cells as mediated by hepatocyte growth factor. *Jpn J Cancer Res* **87**: 702–710

Matsumoto K and Nakamura T (1997) Hepatocyte growth factor as a tissue organizer for organogenesis and regeneration. *Biochem Biophys Res Commun* **239**: 639–644

Miyazawa K, Tsubouchi H, Naka D, Takahashi K, Okigaki M, Gohda E, Daikuhara Y and Kitamura N (1989) Molecular cloning and sequence analysis of cDNA for human hepatocyte growth factor. *Biochem Biophys Res Commun* **163**: 967–973

Morishita R, Nakamura S, Hayashi S, Taniyama Y, Moriguchi A, Nagano T, Taiji M, Noguchi H, Takeshita S, Matsumoto K, Nakamura T, Higaki J and Ogihara T (1999) Therapeutic angiogenesis induced by human recombinant hepatocyte growth factor in rabbit hind limb ischemia model as cytokine supplement therapy. *Hypertension* **33**: 1379–1384

Nabeshima K, Shimano Y, Inoue T, Itoh H, Kataoka H and Koono M (1998) Hepatocyte growth factor/scatter factor induces not only scattering but also cohort migration of human colorectal-adenocarcinoma cells. *Int J Cancer* **78**: 750–759

Nakamura T, Nawa K and Ichihara A (1984) Partial purification and characterization of hepatocyte growth factor from serum of hepatectomized rats. *Biochem Biophys Res Commun* **122**: 1450–1459

Nakamura T, Nishizawa T, Hagiya M, Seki T, Shimonishi M, Sugimura A, Tashiro K and Shimizu S (1989) Molecular cloning and expression of human hepatocyte growth factor. *Nature* **342**: 440–443

Nakamura T, Matsumoto, Kiritoshi A, Tano Y and Nakamura T (1997) Induction of hepatocyte growth factor in fibroblasts by tumor-derived factors affects invasive growth of tumor cells: in vitro analysis of tumor–stromal interactions. *Cancer Res* **57**: 3305–3313

Niederhuber JE, Brennan MF and Menck HR (1995) The National Cancer Data Base report on pancreatic cancer. *Cancer* **76**: 1671–1677

Paciucci R, Vila MR, Adell T, Diaz VM, Tora M, Nakamura T and Real FX (1998) Activation of the urokinase plasminogen activator/ urokinase plasminogen activator receptor system and redistribution of E-cadherin are associated with hepatocyte growth factor-induced motility of pancreas tumor cells overexpressing Met. *Am J Pathol* **153**: 201–212

Parr C, Hiscox S, Nakamura T, Matsumoto K and Jiang WG (2000) NK4, a new HGF/SF variant, is an antagonist to the influence of HGF/SF on the motility and invasion of colon cancer cells. *Int J Cancer* **85**: 563–570

Pepper MS, Matsumoto K, Nakamura T and Montesano R (1992) Hepatocyte growth factor increases urokinase-type plasminogen activator (u-PA) and uPA receptor expression in Madin-Darby canine kidney epithelial cells. *J Biol Chem* **267**: 20493–20496

Poston GJ, Gillespie J and Guillou PJ (1991) Biology of pancreatic cancer. *Gut* **32**: 800–812

Rosen EM, Joseph A, Jin L, Rockwell S, Elias JA, Knesel J, Wines J, McClellan J, Kluger MJ, Goldberg ID and Zitnik R (1994) Regulation of scatter factor production via a soluble inducing factor. *J Cell Biol* **127**: 225–234

Rosen EM, Laterra J, Joseph A, Jin L, Fuchs A, Way D, Witte M, Weinand M and Goldberg ID (1996) Scatter factor expression and regulation in human glial tumors. *Int J Cancer* **67**: 248–255

Rosenthal EL, Johnson TM, Allen ED, Apel IJ, Punturieri A and Weiss SJ (1998) Role of the plasminogen activator and matrix metalloproteinase system in epidermal growth factor-and scatter factor-stimulated invasion of carcinoma cells. *Cancer Res* **58**: 5221–5230

Seki T, Ihara I, Sugimura A, Shimonishi M, Nishizawa T, Asami O, Hagiya M, Nakamura T and Shimizu S (1990) Isolation and expression of cDNA for different forms of hepatocyte growth factor from human leukocyte. *Biochem Biophys Res Commun* **172**: 321–327

Seslar SP, Nakamura T and Byers SW (1993) Regulation of fibroblast hepatocyte growth factor/scatter factor expression by human breast carcinoma cell lines and peptide growth factors. *Cancer Res* **53**: 1233–1238

Shibamoto S, Hayakawa M, Takeuchi K, Hori T, Oku N, Miyazawa K, Kitamura N, Takeichi M and Ito F (1994) Tyrosine phosphorylation of β-catenin and plakoglobin enhanced by hepatocyte growth factor and epidermal growth factor in human carcinoma cells. *Cell Adhes Commun* **1**: 295–305

Sperti C, Pasquali C, Piccoli A and Pedrazzoli S (1997) Recurrence after resection for ductal adenocarcinoma of the pancreas. *World J Surg* **21**: 195–200

Tannapfel A, Yasui W, Yokozaki H, Wittekind C and Tahara E (1994) Effect of hepatocyte growth factor on the expression of E- and P-cadherin in gastric carcinoma cell lines. *Virchows Arch* **425**: 139–144

Uchiyama A, Morisaki T, Beppu K, Kojima M, Nakatsuka A, Mizumoto K, Matsumoto K, Nakamura T and Tanaka M (1999) Hepatocyte growth factor and invasion-stimulatory activity are induced in pleural fluid by surgery in lung cancer patients. *Br J Cancer* **81**: 721–726

Van Belle E, Witzenbichler B, Chen D, Silver M, Chang L, Schwall R and Isner JM (1998) Potentiated angiogenic effect of scatter factor/ hepatocyte growth factor via induction of vascular endothelial growth factor: the case for paracrine amplification of angiogenesis. *Circulation* **97**: 381–390

Vila MR, Nakamura T and Real FX (1995) Hepatocyte growth factor is a potent mitogen for normal human pancreas cells in vitro. *Lab Invest* **73**: 409–453

Warshaw AL and Fernandez-del Castillo C (1992) Pancreatic carcinoma. *N Engl J Med* **326**: 455–465

© 2001 Cancer Research Campaign

Watabe M, Matsumoto K, Nakamura T and Takeichi M (1993) Cooperative action of hepatocyte growth factor and anti-cadherin antibodies on the scattering of keratinocytes. *Cell Struct Funct* **18**: 117–124

Weidner KM, Behrens J, Vanderkerckhove J and Birchmeier W (1990) Scatter factor: molecular characteristics and effect on the invasiveness ofepithelial cells. *J Cell Biol* **111**: 2097–2108

Zarnegar R and Michalopoulos GK (1995) The many faces of hepatocyte growth factor: from hepatopoiesis to hematopoiesis. *J Cell Biol* **129**: 1177–1180

*© 2001 Cancer Research Campaign*
